# *'He is the one who is providing you with everything so whatever he says is what you do'*: A Qualitative Study on Factors Affecting Secondary Schoolgirls’ Dropout in Rural Western Kenya

**DOI:** 10.1371/journal.pone.0144321

**Published:** 2015-12-04

**Authors:** Kelvin Oruko, Elizabeth Nyothach, Emily Zielinski-Gutierrez, Linda Mason, Kelly Alexander, John Vulule, Kayla F. Laserson, Penelope A. Phillips-Howard

**Affiliations:** 1 Kenya Medical Research Institute (KEMRI), Center for Global Health Research, Kisumu, Kenya; 2 Center for Global Health, Centers for Disease Control and Prevention, Atlanta, GA, United States of America; 3 Department of Clinical Studies, Liverpool School of Tropical Medicine, Liverpool, United Kingdom; University of Massachusetts Medical School, UNITED STATES

## Abstract

Education is an effective way to improve girls’ self-worth, health, and productivity; however there remains a gender gap between girls’ and boys’ completion of school. The literature around factors influencing girls’ decision to stay in school is limited. Seven focus group discussions took place among 79 girls in forms 2 to 4 at secondary schools in rural western Kenya, to examine their views on why girls absent themselves or dropout from school. Data were analysed thematically. Lack of resources, sexual relationships with boyfriends, and menstrual care problems were reported to lead directly to dropout or school absence. These were tied to girls increased vulnerability to pregnancy, poor performance in school, and punishments, which further increase school absence and risk of dropout. Poverty, unmet essential needs, coercive sexual relationships, and an inequitable school environment collude to counter girls’ resolve to complete their schooling. Lack of resources drive girls to have sex with boyfriends or men who provide them with essentials their family cannot afford, such as sanitary pads and transport to school. While these improve quality of their school life, this dynamic increases their exposure to sexual risk, pregnancy, punishment, and dropout. Evaluation of interventions to ameliorate these challenges is warranted, including provision of pocket money to address their needs.

## Background

Education is an effective way to improve girls’ self-worth, health, productivity and to foster participation in civil society [1]. Staying in school has been shown to protect girls against early marriage, teen pregnancy, and HIV infection, with schoolgirls reporting less frequent sex, fewer partners, and reduced age disparity [2–5]. Evidence has also accumulated on the economic advantages of girls education which improves GDP per capita, women in the workforce, infant and child survival, and the education of future generations [6]. Confirmation of such associations has strengthened international resolve to identify interventions to improve educational opportunities for schoolchildren, particularly girls [7]. Much of this focus has been health-oriented [8].

Among the six Education For All (EFA) goals significant progress has been made on achieving universal primary education, and gender parity at primary levels over the past decade [1,7,9,10]. However, enrolment achievements have stalled in Sub-Saharan Africa (SSA), with a rise in out-of-school children between 2008 and 2010, with larger gaps outstanding on the retention of children through secondary school, and reaching gender parity [1,7,9,10]. The UN estimates 34 million adolescent girls (lower secondary age) are out of school, with the widest gender gap at secondary school level [7,9]. Of 19 countries classed as having ‘severe disadvantage’ for girls education (gender parity index below 0.90), due to poverty, preference to educate boys, and societal pressures, 12 are in SSA [9].

In Kenya primary education has been free since 2003, with the Kenyan government (through the Kenya Education Sector Support Programme) extending free education into secondary schools in 2008, with an annual allocation approximating Ksh10,500 (~£66) per student to cover school tuition fees [11], although additional levies charged to parents are not included. The gender gap between boys’ and girls’ primary school completion narrowed to a 0.98 gender parity index (GPI) in 2008, although girl completers dropped from 49% to 47% [12,13]. Only 58% transitioned to secondary school with the GPI decreasing to 0.92, as fewer girls reached secondary level [13]. One study estimates 64% of girls dropout at secondary school level [14], with the remaining girls less likely than boys to achieve C+ or above in national exams [13,15]. In order to retain girls in school and redress the gender imbalance, reasons behind these phenomena need to be understood. A small body of literature has explored reasons for non-attendance of girls in Kenyan schools, although most have concentrated on primary school with a variety of contributing factors cited including the geographical environment, financial or social issues, and health. A study in low-income areas of Nairobi concluded that the school levies still charged in school kept children out of primary school [16]. Further evidence highlighted gender discrimination in how finance was apportioned–girls with male siblings received less money for fees, and were sent home for non-payment, while their brothers remained in school [17]. Discrimination was further demonstrated with a general lack of concern for their daughters’ welfare which affected schooling in a number of ways such as lack of basic needs, sanitary ware and food, as well as paraffin to provide lighting for completing homework [17]. In contrast, pregnancy and marriage were described as the main cause of dropout in another study [15]. In a similar vein, dropout before secondary school completion is, in part, explained by girls’ vulnerability once they engage in premarital sex, seen as a precursor to marriage [3], with an association found between lack of an equitable environment in school and sexual behaviour among schoolgirls fuelling the risk of pregnancy and dropout [18,19]. One recent report cited physical access, school fees and out of pocket expenses as key contributors to keeping girls out of secondary school, while poor quality of primary schooling was seen as a barrier to enrolment [13]. More recently qualitative studies in primary schools suggest that menstruation contributes to school absenteeism, possibly extending to school dropout [20–22], although this has not been confirmed in secondary schools. The present study was therefore undertaken to provide further understanding about why the school gender gap remains. The study aimed to explore factors that influence girls’ ability to stay at school, and what causes them to absent themselves, or to dropout from secondary school.

## Methodology

### Study Area and Population

The study site was located in Gem sub-county, Siaya, in the former Nyanza Province of western Kenya. The population are commonly members of the Luo ethnic group, who are mainly subsistence farmers and fisher-folk [23]. While Nyanza has a literacy rate of 70% compared to the national average of 62%, surveys estimate four in ten child learners miss school daily [24]. The population is included within a health and demographic surveillance system (HDSS) [25]. A menstrual solutions feasibility study had been conducted among primary school girls in the same area [26].

### Recruitment and Participants

Four out of 21 secondary schools in the study site were purposively chosen, representing mid-sized, rural, secondary schools. All were co-educational and day schools. Forms two to four were chosen in order to target girls aged between 16–18 years. The local Ministry of Education gave permission to undertake the study as did the school head teachers following discussion about its’ intended purpose. The FGD team then gave a talk to girls in the chosen forms and invited them to participate. All girls reported a willingness to do so, with no refusals. Therefore a teacher and the school head girl were asked to blindly select names, picking four girls randomly per year from the registers to give a group of 12 for each FGD. Once participants were selected, field staff visited girls’ parents in their homes to explain the study and ask permission for their daughters’ participation. Girls were only included if written informed consent was received from the parent. Written informed assent was then obtained from the girls at the beginning of the focus group, as per the ethical committee requirements. FGDs were held in a private room on the school compound.

### Focus Group Approach

A semi-structured FGD guide was developed to explore factors that influence girls’ ability to stay at school, what causes them to absent themselves, or dropout, and to understand the wider context in which these occur. We conducted a FGD in three of the four schools to iteratively test the guides (one was unavailable) in August 2013. In January 2014 a second FGD was conducted in the four schools, with new participants to augment the data and to explore in further detail the initial findings. Each FGD began with discussion of ground rules which included the need for confidentiality, to respect each other’s opinions, and to emphasise there were no ‘right’ or ‘wrong’ answers. An ‘ice breaker’ was used to put participants at ease and encourage them to be vocal. The FGDs lasted between one and a half to two hours, were audio-recorded, and were conducted in English, Kiswahili or Luo, as preferred by the group. The moderator and note taker, who worked across all FGD’s were trained local residents and fluent in all three languages. Notes were made to capture main points, group dynamics and non-verbal gestures. The discussions were transcribed by the moderator or note-taker; any Luo/Kiswahili was translated into English. The transcripts were reviewed while listening to the recording and checked against notes for accuracy. For each quote, a code was used to define the school, and when the FGD took place. P (number) was used to represent the participants, with Px denoting when a participant number was missed in note taking.

### Analysis

Lead authors (KO, EN, and PPH) familiarized themselves with the transcripts before entering data into NVIVO (version 10). Coding of themes was performed as themes emerged. Codes were assigned to the abstracted sections with authors comparing key themes until consensus was reached. Each section of data representing a specific code was reviewed for relevancy. Text was un-coded and reassigned to different or new code in situations of disagreement or where a new, more precise code was developed that provided a better fit to the data. These themes were then developed using girls’ narratives interwoven with text into the draft manuscript, reviewed by LM, KL, EZG, and KA.

### Ethics

Ethical approval was granted from the Scientific and Ethical Review Boards of the Kenya Medical Research Institute (SSC No 2198), the Institutional Review Board of the U.S. Centers for Disease Control and Prevention, and the Ethics Committee of the Liverpool School of Tropical Medicine (12.11).

## Results

Seven (7) FGDs were held comprising 79 girls in total, with 11 or 12 girls per group. The majority (69%) of participants were 16–18 years old, with 17% below this age, and 14% older. Girls were spread equally across each year (forms 2–4).

The content of all FGDs were strikingly similar, with comparable themes emerging across all discussions suggesting that data saturation was largely reached. Participants described both direct causes of dropout, as well as causes of absenteeism which may culminate in dropout. These have been grouped into 10 themes: menstruation, school expenses, gender imbalance, competing demands, poor academic performance, punishment, relationships, obligatory sex, and pregnancy. However, there was considerable overlap between these themes which were often part of a cascade of events culminating eventually in dropout. While lack of resources was the key driver behind many of these themes there were also emergent subthemes of embarrassment, girls’ vulnerability and peer/male/parental pressure.

When asked if their female peers dropout from school, there was generally a chorus of ‘Yes’ by all participants, indicating this was a well-accepted phenomena. The moderator, having ascertained that this was so, then focused the discussion around the reasons why. In all FGs the same range of reasons were proffered, however in 5 out of the 7 FGDs the first response attributed absenteeism to menstruation, while it was given as the second and third reason in the 2 remaining FGDs. Absence due to menstruation was mentioned by participants themselves as well as reports it occurred amongst their peers.

*‘I think girls like me when I am having my menses I do not always feel comfortable coming to school*. *So I can absent myself from school*, *at least for a day*, *then the following day I come’* (P3, School 4b)

Commonly the following situations were described as arising from poor menstrual protection; impaired concentration, embarrassment, the need for frequent latrine visits to check leakage, restricted movement and the need to leave school to wash or change stained clothing. Sometimes girls returned to school once changed, other times they remained at home until the following day or when their menses stopped. Girls spoke of having to use cloths, pieces of mattress, tissue paper, and cotton wool to manage their menstruation when sanitary pads were unaffordable to them. They described the cumbersome and non-absorbent properties of these materials which resulted in pain, rashes, leaks, soiled clothes, bad odour, and consequent teasing by classmates.

*‘You are like in class and then…when the blood is coming out*, *so it went until to the cloth and desk*, *… then when I stood a fellow told me I have stained’* (P9, School 4b).

The fear or embarrassment of having stained clothing which forced girls to remain at home until menses ceased was echoed throughout all groups. However, there was some disagreement as to whether this led to dropout or only absenteeism.

‘*Monthly period is foremost (reason) that girls dropout* (P7, School 1a).

‘*It cannot cause dropout because it happens for 3 days only and it’s gone’* (P5, School 1a).

*‘I think it can led to dropout because each and every time you absent yourself from school*, *your performance will be poor and you will decide to drop out of school’* (P7, School 1a).

Pain and menstrual cramps were also mentioned as a reason for absenteeism but to a much lesser extent suggesting the impact on schooling was minimal.

Lack of school fees was also described in all FGDs as a major contributor to girls absence from school, and was also a direct reason for school dropout. The lack of resources also had an impact on school attendance not just because of inability to pay school fees, but also the ability to purchase school essentials such as school uniform and books. It was widely reported that dropout was far higher among girls than among boys. The reasons given were that when resources were scarce, parents would pay school fees for their sons but not their daughters, with participants stating:

*‘In this community they still believe girls are not supposed to be educated’* (P7, School 1a).

‘*Girl child is still not recognised*, *not given opportunity to learn; their siblings who are boys are given first opportunity’* (P11, School 3a).

The consensus was that education was seen as less important for girls as they would be married off in the future. Girls commonly described that they, or their peers, were also kept back from school to complete chores, earn money, and look after their siblings while their parents worked, or—as mentioned occasionally—because they were orphans:

*Now you see things to eat at home is not there*, *so it will force this girl to work for someone in the shamba (garden)*. *After digging she gets a hundred and fifty*, *take it then use it to buy some flour*. *That day she has gone to the shamba she will miss school*. *Yes I will go to the shamba today*, *tomorrow I go to school*. *The following day I go work again to get some money for food*. *You see they cannot manage the kind of life they are living*, *so they are forced just to do any work to get the money for food*. *It is like they are the ones managing those families* (P11, School 1b).

*‘To me*, *most of the time my parents are not at home*, *so I am the one who is responsible to take care of our young siblings*, *so sometimes (it) is forcing me not to come to school’* (P9, School 4b).

*‘Most of them when parents have died they stop coming to school because their guardian they are left with does not care*, *so maybe they just have to do what they can*. *Maybe selling bananas so they can feed the other siblings*. *So they cannot come to school’* (P11, School 1b).

Frequently girls reported that they, or their peers, had to juggle school and household chores; undertaking chores late at night caused tiredness and prevented completion of school assignments. Doing chores first thing in the morning also led to fatigue, resulting in lack of concentration, occasional dozing in class and sometimes resulted in punishment. The long distances some girls had to travel to get to school (one group mentioning this may involve walking for one and half hours), compounded this problem. It also sometimes led to girls being late for school, which again could lead to punishment. Sometimes, to avoid this, girls simply stayed at home that day.

‘*For you to [do] all these things [wash utensils*, *prepare supper*, *clean uniform] you may finish at around ten in the night…and you are tired*, *because you have also walked a long distance*, *so you need to go and rest… because you have slept late…by six*, *it will not be possible for you to do the assignment’* (P2, School 1b).

Others described taking lifts to or from school from boda-boda motorbike drivers if they were tired or late because of doing their household chores, to avoid the consequent harsh punishment at school. The drivers wait for vulnerable girls close to their homes or around the school, initially giving the trips free of charge; girls recognised they can use such offers occassionally in an emergency but if they accepted a lift more frequently ‘*it is not for free’*.

*‘If you are not able to give money it will force you to have sex with them’* (P1, School 1b).

Poor performance in school was also associated with a lack of resources, such as money to buy kerosene, which girls need to study or complete assignments at night or early in the morning.

*‘Well mostly some families are poor*, *and they use the small kerosene lamps*, *and when there is no kerosene the student will not be able to do the assignments or when she leaves early to school*, *the lights are not there*, *there is black out*, *she cannot do the assignments’* (P11, School 1b).

Participants in each FGD concluded that the poor performance caused some girls to withdraw from school, either because they were disillusioned and shamed by having continually poor results, occasionally reaching breaking point when teased by peers, or they were simply unable to catch up. In one school, girls said test results are announced in assembly, so everyone is aware who is at the bottom of the class. The humiliation faced by these girls facilitates their dropout. A couple of girls mentioned that no system was in place to help them catch up after an absence, although others spoke of having friends help them understand lessons they had missed.

Girls from poor families may be late-aged entrants or may re-sit grades due to poor performance, or.d absence resulting from lack of fees or home commitments. This places pressure on older girls who feel humiliated by younger girls who ‘*start calling you mother’* a phenomena described in just one FGD. In a couple of instances participants stated that teachers also embarress older girls by refering to their age.

‘*When you make simple mistakes they comment—like your agemates finished school long time ago’* (P9, School 3b).

Poor performance was reported to be a breaking point for some girls teased by their peers. Others who were struggling saw no benefit to staying in school, since working would at least bring benefits to the household:

‘*There are girls who do not perform*, *she keeps being last each and every time*, *so she prefers not to go to school and prefers to work (instead)’* (P1, School 1b).

The system of punishments described in six of the FGDs was also seen as responsible for school absenteeism and occasional dropout. If school rules were breached (one example cited was taking a second lunch serving due to hunger), girls are punished by having to bring food or other (compensatory) items, such as barbed wire from home. Dropout occurred if families could not afford these goods, yet again the lack of resources was cited as a driving force for absenteeism:

‘*She will not come back to school because this person (the girl) was double served*.*so where is she going to get a sack of maize and (of) beans while they do not have food at home’* (P1, School 1b).

Girls reported that harsh punishments doled out by teachers discouraged girls from attending. These included sweeping the compound, slashing, picking rubbish and being caned often on the buttocks which girls in one group found shameful particularly when they were menstruating. Participants in one FGD ranked cleaning the latrine of faeces as the most embarrassing punishment. One participant described being ‘*given a blow or knock out by the teacher’*. Being watched by friends or boyfriends increased the embarrassment suffered by such girls as friends or peers mocked, branding them with humiliating names such as ‘s*chool mopper’*. The punishments were viewed as particularly hard when the reason for absence was ‘*genuine*’ such as having to look after siblings, or illness.

*The teacher do not understand like there are some things that girls cannot do*, *girls do get tired very quickly*, *they cannot slash for two hours like boys*. *Sometimes you are in your periods and you are given a larger portion to slash* (P3, School 2b).

Peer, parental pressure and relationships with boys were other causes of absenteeism and dropout, cited in all FGDs. Yet again, this was also linked with lack of resources. The majority of participants declared they and their peers had boyfriends. Many acknowledged that having a boyfriend caused girls to lose interest in schoolwork, resulting in a *‘failure to perform’* among some who preferred to spend time with the boys instead of studying. However, peer pressure to have a boyfriend was apparent:

*If they know you don’t have a [boy] friend they will abuse you that you aren’t normal’* (P10, School 3b).

One girl explained that some boyfriends felt threatened if a girlfriend was educated:

*‘A girl has a lover who is a campus guy*, *and you know some guys nowadays do not want to marry girls who are learned*, *because they are well educated and will be a competitor*, *so it will force the girl to get into marriage early and not continue with the school’* (P9, School 4b).

Some girls considered studying was irrelevant as they will get married and not need to be educated, although this was suggested to be the thoughts of ‘others’, not the FGD participants. One expression was ‘*someone is studying for them–their future husband*’. This view was not unanimous, however, with other girls saying boyfriends were very supportive. One girl stated ‘*if my boyfriend really loves me he will want me to finish school’* (P2, School 3a).

Participants reported that a lack of essential items or small luxury items influenced their decision to remain in school or dropout. An inability to buy essential needs within the family budget forced girls to seek money or goods from boyfriends. In all focus groups, narratives described how some impoverished parents would encourage their daughters to obtain items through sex.

‘*There are parents who when you ask for money for pads they tell you*, *you are a big girl*, *so you use the money from boyfriends to buy pads* (P4, School 4b).

Girls themselves also recognised the need to have sex for money to purchase essential basic items. Sanitary pads were commonly cited; other items include panties, toiletries like body lotion, face cream and Vaseline™, food or drink.

*‘Because parents cannot provide you with everything that you want like pads hence you will have to indulge in sexual intercourse’* (P7, School 2a).

Girls seemed resigned to this, describing feeling or being obligated to have sex for essentials.

*‘Most of the time the one who decides (for sex) is the boy because he is the one who is providing you with everything so whatever he says is what you do’* (P2, School 1b).

Boda-boda drivers were also a source of cash to buy essentials ‘*he can give you 100 (~£0*.*65) shillings to buy pads’*, with some girls actively asking them out to get money, but again, the drivers would demand sex in return:

*‘He can give you 200 shillings (~£1*.*30) to buy your own things or maybe drink soda*, *so girls after getting this money they feel that they are on top of the world‘* (P10, School 2b).

It appeared that some schools try to prevent girls having relationships with boys by adopting strict punishments. In both FGDs held at one school, girls said the school held a fake wedding for a boy and girl found to have a relationship, with the couple ‘*wedded at the assembly ground’*. The pupils would then be suspended. The girls felt so ridiculed and humiliated by the fake wedding in front of the school they would then absent themselves, transfer to another school, or dropout entirely.

When asked what the most important reason was for girls dropping out of school, the most common response from all groups was pregnancy. However, it was not clear whether girls chose to dropout or were expelled, as contradictory accounts were provided.

*‘It is illegal to be pregnant in school* (P7, School 4b).

‘*Pregnancy is the most important because they (girls) are shy they move away and leave school’* (P1, School 3a).

One participant remarked that even if parents tried to keep their daughter in school, she would be too shamed by the gossip to remain. The shame was heightened by the predominance of male teachers whom girls commonly considered to be lacking in empathy.

*‘There are some parents who will share with the teachers that nowadays you teach pregnant girls in school*. *So they will discuss this issue with the teachers*. *We do not have madam teacher to share with such problems*, *so the male teachers will discuss the issues and the whole school will come to know that you are expecting and therefore you will just dropout of school* (P3, School 2b).

Most acknowledged pregnancy was accidental, with a lack of sexual knowledge among girls of ‘*a tender age they do not know when they do it [sex]’*. Interestingly, there was some difference of opinion regarding being pregnant whilst still at school:

*‘You know God created them to fill the earth*, *therefore it is not wrong for girls to be pregnant in school’* (P12, School 3b).

*‘It is wrong for girls to be pregnant in school because sometimes your parents have struggled to pay for your school fees and maybe you reached form three and you are pregnant*. *So now you will leave without the certificate therefore it is like you wasted your parents’ money’* (P1, School 3b).

Pregnancy was considered the endpoint in a cascade of events driven by poverty, resulting in dropout:

‘*Some of them [girls] because of lack of pads*, *because their parents cannot manage to buy them what to use*, *they go to other people like boyfriends to buy them*, *sometimes they have sex and by that time she is going to drop out due to pregnancy’* (P4, R2, School 1).

Getting married was not viewed as a direct cause of dropout amongst our participants, however, with just a single instance reported in the FGDs. Marriage occurred because of pregnancy, or as a consequence of poverty, but did not prevent girls from staying at school, with girls noting some peers secretly married (while at school) to working men, including teachers, to improve their quality of life:

‘*You become his wife secretly*. *He will be paying for you(r) school fee*, *and so those many odd jobs you used to do*, *you now stop’* (P1, School 1b).

## Discussion

This study follows on from a range of qualitative and quantitative studies demonstrating girls’ absenteeism and dropout from school, preventing them from reaching their academic potential and future productivity [6,9,14], and impacting their health and wellbeing [2–5]. Preventing dropout has an impact beyond the individual; one economic modelling study in Kenya estimated that if 1.6 million girls complete their education and the 220,098 adolescents became employed rather than falling pregnant, the cumulative effect would add US$ 3.4 billion to Kenya’s annual gross income [14]. While numerous studies reflect on whether pregnancy and marriage are the main cause of schoolgirl dropout in SSA countries [15,18,19,27–32], girls in our study described contextual factors that increase girls’ exposure to pregnancy, although not marriage, which force them to dropout. Girls’ narratives mapped a path of cascading events often initiated by poverty but abetted by unequal and punishment-oriented environments. This often resulted in an inability to meet personal or academic needs, causing them humiliation and shame, which girls alleviate by seeking help from boyfriends, obligating sex and raising their risk of pregnancy or loss of interest in school. While such descriptions are nuanced, the underlying context is one of dependence on boys and men as a social norm, with some parents encouraging this to reduce the family’s burden. The complex interlinkage so evident in the present study between poverty, peer influence, sexual risk taking and pregnancy—resulting in dropout—has been noted elsewhere [15,28].

Poverty was a constant underlying element in girls’ narratives [11], and responsible either directly or indirectly for the greater proportion of absenteeism or dropout. Lack of cash for fees or personal needs was noted as a main factor directly leading to dropout. Although the Kenyan government extended free education from primary into secondary schools in 2008, girls’ narratives describing ‘school fees’ drove us to examine what comprised ‘fees’ in the area since tuition fees were already paid. Schools provided us with standard invoices per student; these included PTA / development levies, and fees for lunch, exams, activities, PE, district evaluation, and ICT maintenance. Adding further costs for uniforms and books, we estimate around Ksh 20,000 (~£126) is required per girl each year. A separate study suggested these additional costs amount to 55% of the average rural households’ annual per capita expenditure [13]. Should such fees be afforded for a girl (assuming no competing male sibling), any further personal items required by girls would be beyond the reach of many families who have a mean wealth index of ~£426 [33]. We noted two-thirds of the annual fees are requested in the first school term. Girls’ dropout over the year thus occurs after the bulk of fees are paid, with a lack of smaller sums of money acting as a barrier to completion.

Girls in our study describe many items they need that are beyond the household budget. Top among these were sanitary pads for menstrual care. The reporting of menstrual care problems in school was very common in the present study, with girls discussing this as a main cause of school absence, and some considering repeated absence resulted in eventual dropout. Teasing and public humiliation, discomfort from poor quality sanitary items, and poor engagement due to fear of leakage and odour are well described in the literature [20,21,34]. Sanitary pads are a frequently cited item girls say they need, but parents’ budgets seldom stretch to provide the quality or amount required. Girls’ narratives indicate some boyfriends provide pads directly, while other girls request money from sexual partners to buy pads. In a study among girls and women below 30 years of age in the same area, half said they received commercial pads from a sexual partner [35]. While sex for money in order to purchase pads was seldom recorded (~3% among young single females), it was reported among 10% of 15 year olds. It was significantly higher among girls dependent on family for source of income, and among girls reporting two or more sexual partners in the past 12 months [35]. Removing menstrual need as a burden for schoolgirls would improve their dignity, engagement in school, and potentially improve their performance. It would also reduce one reason for dependence on sexual partners, and could contribute to reducing their exposure to sexual and reproductive harms, and possible school dropout.

Along with menstrual items, a variety of relatively low budget items such as face cream, body oil, panties, sodas, and snacks were also seen as necessary items for girls to have. Having a boyfriend was considered essential, as boys (and men) buy them or provide money for these needs. A strong literature exists on procurement of socially desirable items from sexual partners in impoverished communities [36–38], with a particular focus around implications for HIV risk for young females [39,40]. Some research shows value is placed on receipt of gifts as a ‘token of appreciation’ and respect of partners [36,37], while other studies show ‘transactional sex’ provides equity in the relationship [41]. Transactional sex appears to be accepted and appreciated by older generations as a way of showing worth to young females [37,41], and is considered routine and not coercive [42]. Studies also comment on material and monetary expectations from committed partners [41]. In our study we are reticent to consider girls’ receipt of basic essentials for obligatory sex as ‘transactional’, as the narratives largely illustrate girls feeling forced or obliged to have sex. Girls without boyfriends felt peer pressure to conform, and desired to have the items that they see other girls having access to. While most girls have boyfriends, and some girls receive items from them, certain groups, such as boda-boda drivers and older men, clearly prey on girls who are particularly vulnerable because of the punitive and humiliating punishments arising if they do not adequately juggle school, homework and house chores.

Girls in the present study area are raised in an environment endemic to physical, sexual and emotional violence; in the nationally representative Kenyan Demographic Health Survey, 25% of women in Kenya reported violent abuse in the 12 months prior to survey (KNBS) [43]. Highest rates are reported in Nyanza Province, where this study was conducted; here 36% reported physical violence in the past 12 months, with 32% reporting lifetime sexual violence (KNBS) [43]. Girls in our study describe teasing and humiliation as a norm, with different punishments inflicted on them in school, including physical violence, public humiliations, and sexual harassment. Such punishments take place in front of peers and boyfriends, increasing their shame and decreasing their motivation to remain in school.

A separate study in Kenya highlighted that a poor learning environment, gender-unfriendly schools, and sexual harassment increased girls’ likelihood of school dropout [18,31]. In Sierra Leone girls felt female teachers were friendlier than the males who sexually harassed them in school [44]. Girls in our study said their fear of punishment increased with the predominance of male teachers who they say lack empathy for girls, with sexual overtones, and breach confidentiality on pregnancy. Male teachers also fail to appreciate the burden of work girls face outside school hours and humiliate them when they fall behind in class. Such discouragements further influence girls' decision-making on whether to remain in school.

As well as lacking school fees and essential items, poverty requires girls to perform domestic labour in tandem with attending school, impacting on their school participation and school attainment [45]. Statements by girls illustrate that the burden of domestic chores falls on them rather than their male siblings. Despite the greater burden, however, girls’ workloads when at school is lower than their same aged girl counterparts who left school [46]. Girls are required to balance household activities and school work, with narratives describing that these obligations result in tiredness, an inability to concentrate in class, and non-completion of school assignments [47]. Girls describe this hampers their performance in school, leading to humiliation in class and punishment, lethargy towards engagement in class, absenteeism and, for some, eventual dropout. Girls in a study conducted in Nigeria ranked poor performance as the second major reason for dropout after poverty [48], with poor performance and grade repetition associated with pregnancy-related dropout in South Africa [27]. Loss of interest in schoolwork was also reflected in some narratives about boyfriends and peers who reinforced perceptions that girls who leave school can earn money or be supported through marriage. It was encouraging to hear some girls in our study demonstrate their determination to succeed in school ‘*for their future’*.

Pregnancy is both considered and contested as the leading cause of dropout among girls in SSA [15,18,19,27–32], yet again in the present study it was at least in part, described as a consequence of poverty through the mechanism of transactional sex. Some authors consider it an international crisis affecting the socio-economic welfare of countries, societies and families at large [49], while others caution that pregnancy is not the main cause of dropout, and other sociological factors need to be considered [18]. In our study many narratives considered pregnancy an inevitable endpoint in the causal pathway to dropout. Pregnancy is clearly viewed as a stigma to the girl and the family [30], leading to exclusion or expulsion from school despite a statute to retain pregnant girls in school [15]. It is estimated one in five adolescent girls have a live birth before 18 years of age, the majority of these are concentrated in south Asia and SSA, with the greatest increase in the next 20 years predicted to be in SSA [50]. Impoverished girls living in remote, rural areas are at greatest risk. There is a vast international literature around pregnancy prevention [51]. In SSA, keeping girls in school is a foremost strategy to prevent pregnancy [51]. A South African study found an association between school performance, grade repetition, temporary withdrawal and girls’ likelihood of pregnancy during school, resulting in pregnancy-related withdrawal and long term dropout [27]. Literature suggest parental monitoring and personal self-efficacy could delay child-bearing until older [32]. We detected a number of girls reporting parental absence or neglect, causing girls additional stress and hardship suggesting that evidence from the literature would not be transferable in the present context. A Ugandan study demonstrated girls’ fears of harsh confrontation and disappointment of parents once pregnancy was detected [52]. Lack of sex education and access to reproductive health services is clearly a problem; in our study, girls reported few of their peers used contraceptives; with the majority of participants voicing suspicion that contraceptives caused infertility, barrenness, and deformities. Sadly, while girls continue to lack education and resources and the prevailing attitude towards transactional sex is one of acceptance and normality, it is unlikely that this situation will change.

At the end of each FGD we asked girls what could be done to keep them in school. Provision of sanitary pads was most often requested, with some suggesting financial support from bursaries and sponsors, and many other girls asking for pocket money. The latter would pay for small luxuries (panties, snacks), school books, food, as well as sanitary pads. Provision of disposable pads is expensive; however, a feasibility study has shown reusable menstrual cups may be of benefit in this setting and deserves further study [22]. Girls also requested more female teachers and a trusted female presence in school for discrete counselling. Better education on sexual and reproductive health and life skills was suggested, as was finding ways to educate their parents. Remedial education programmes to allow catch up after absence would clearly benefit girls also [13]. Cash transfers to parents, conditional upon reaching specific attendance targets are considered a way to bolster girls attendance [13], and reduce child labour [45]. These have been shown to have a direct impact on girls sexual and reproductive health, with relatively small sums (of ~US$5/month) recommended [4,53]. A recent review found while both conditional and unconditional cash transfers improved the odds of enrolment and attending school (compared with none), conditional cash transfer was significantly more effective when conducted under a strict monitoring and penalty system [54]. We suggest testing the value of cash transfer to girls, with close monitoring to evaluate how it is spent and what social pressures occur.

We recognise a number of limitations in this study. There may have been desirability effect through introducing the moderator and note keeper, with girls wanting to provide information to please these outsiders. We attempted to minimise this by having young female moderators and note-keepers from the same local area, but who did not personally know the girls. It was noted that the opinions were similar not just within the groups but across the groups, suggesting that to a large extent the views represent many if not all individuals who participated in the FGDs. Our previous study among primary schoolgirls was used to iteratively develop the guide, which evolved between the initial and later focus groups. Secondary school girls in the initial focus groups may have broadcast their discussions before the later ones, thereby influencing girls’ responses; however, we minimised this by discussing the importance of privacy among the girls, and by including different participants each time. Girls’ claims about male teachers’ inappropriate behaviour and other men’s coercion could not be individually verified but the narratives were similar across FGDs suggesting these concerns were commonly perceived.

In summary, our study confirms and adds to a small literature illustrating the challenges girls face while remaining in education in a rural SSA setting. Poverty, unmet essential needs, coercive sexual relationships, and an inequitable school environment collude to counter girls’ resolve to complete their schooling. Evaluation of interventions to ameliorate these challenges is warranted.
